# Supplementary material for: Enhanced Processivity and Collective Force Production of Kinesin-1 at Low Radial Forces
Source: bioRxiv. 2025 Nov 22:2025.08.27.672644. Originally published 2025 Aug 31. Preprint. [Version 2] doi: 10.1101/2025.08.27.672644 (PMC12407981; doi:10.1101/2025.08.27.672644)
Supplement: 1 [file NIHPP2025.08.27.672644v2-supplement-1.pdf]

bioRxiv preprint doi: https://doi.org/10.1101/2025.08.27.672644; this version posted November 22, 2025. The copyright holder for this preprint (which was not certified by peer review) is the author/funder, who has granted bioRxiv a license to display the preprint in perpetuity. It is made available under aCC-BY 4.0 International license.

Supplementary Figures

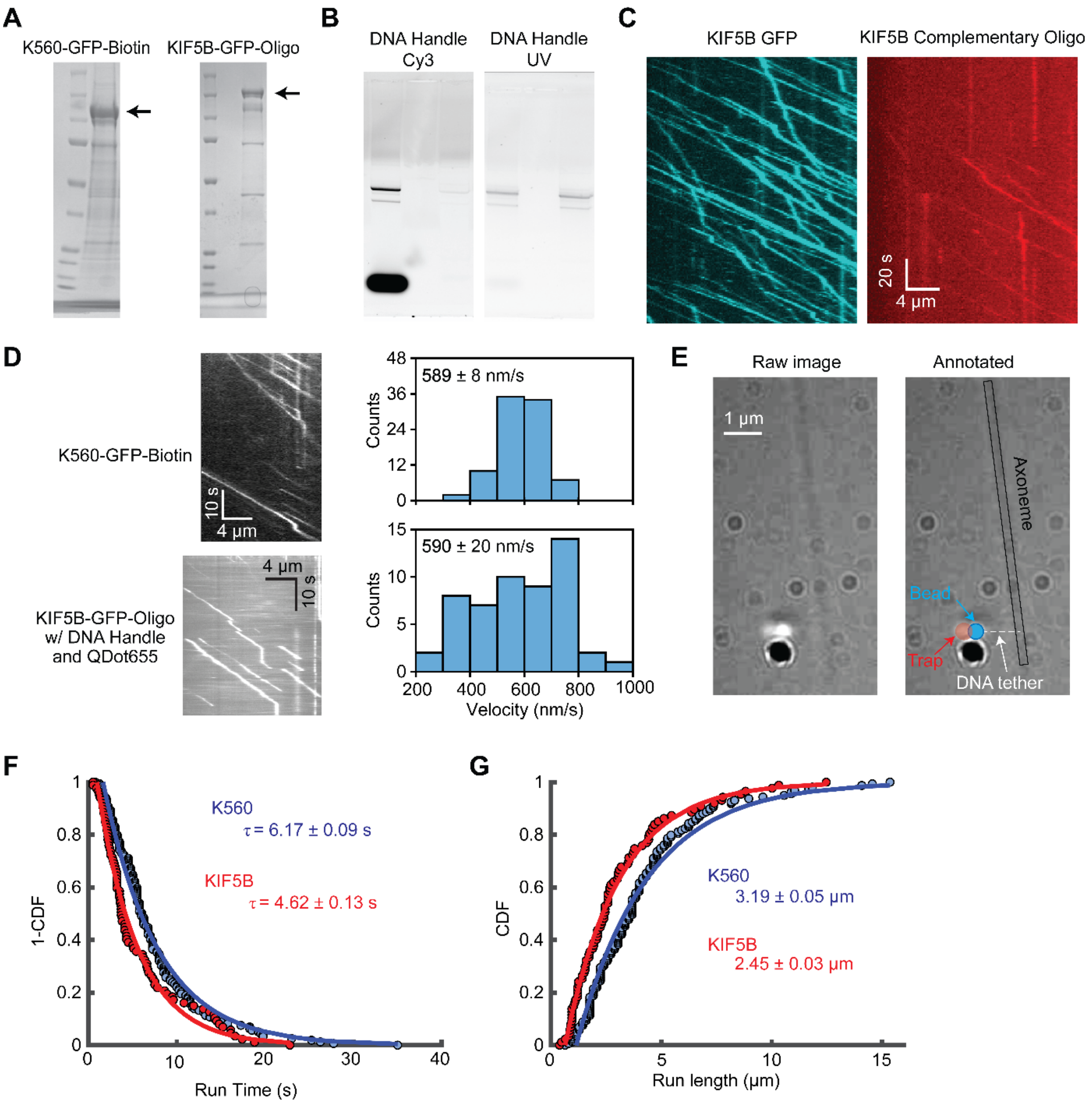

Figure 1_figure supplement 1. Purification of kinesin and DNA handle with experimental controls. A) SDS-Page denaturing gel of constructs used. K560-GFP-SNAP was labeled with biotin, and KIF5B-GFP-SNAP was labeled with a BG-functionalized DNA oligo before eluting the motor from the IgG beads during purification. **B)** The long DNA handle was run on a 0.8% TAE agarose gel. Left: Handle incubated with a 20-fold excess of complementary Cy3 oligo and imaged in the Cy3 channel of a Typhoon FLA 9500 fluorescence imager. Right: The same gel imaged under UV after staining in 2x GelRed for 40 minutes. **C)** Full-length KIF5B-GFP-SNAP was incubated with a 10-fold excess of complementary Cy5-labeled oligo and run in a motility assay. The assay was performed in the presence of 10 nM MAP7. **D)** Kymographs of K560-GFP-

bioRxiv preprint doi: https://doi.org/10.1101/2025.08.27.672644; this version posted November 22, 2025. The copyright holder for this preprint (which was not certified by peer review) is the author/funder, who has granted bioRxiv a license to display the preprint in perpetuity. It is made available under aCC-BY 4.0 International license.

biotin (top, N = 89) and Qdot 655 streptavidin quantum dots being transported by oligo-labeled KIF5B attached to the long DNA handle (bottom, N = 53). Histograms of the respective velocities are shown to the right. **E)** Mechanical demonstration of a bead tethered to an axoneme by the long DNA handle. After tethering to an axoneme, the bead is pulled away from the axoneme by an optical trap. The bead moved freely for ~1 µm and stayed in that position, demonstrating the formation of a long tether between the bead and the axoneme through the DNA handle. **F)** 1-CDF kinesin run time under unloaded conditions in single-molecule fluorescence imaging assays (N = 160 for K560 and 88 for KIF5B). Solid curves represent a fit to a single exponential decay function to calculate the lifetime of processive runs (τ, ±S.E.). **G)** CDF of kinesin run length under unloaded conditions in single-molecule fluorescence imaging assays. Solid curves represent a fit to a single exponential decay function to calculate the mean run length (±S.E.). In D, F, and G, KIF5B assays were performed in the presence of 50 nM MAP7.

bioRxiv preprint doi: https://doi.org/10.1101/2025.08.27.672644; this version posted November 22, 2025. The copyright holder for this preprint (which was not certified by peer review) is the author/funder, who has granted bioRxiv a license to display the preprint in perpetuity. It is made available under aCC-BY 4.0 International license.

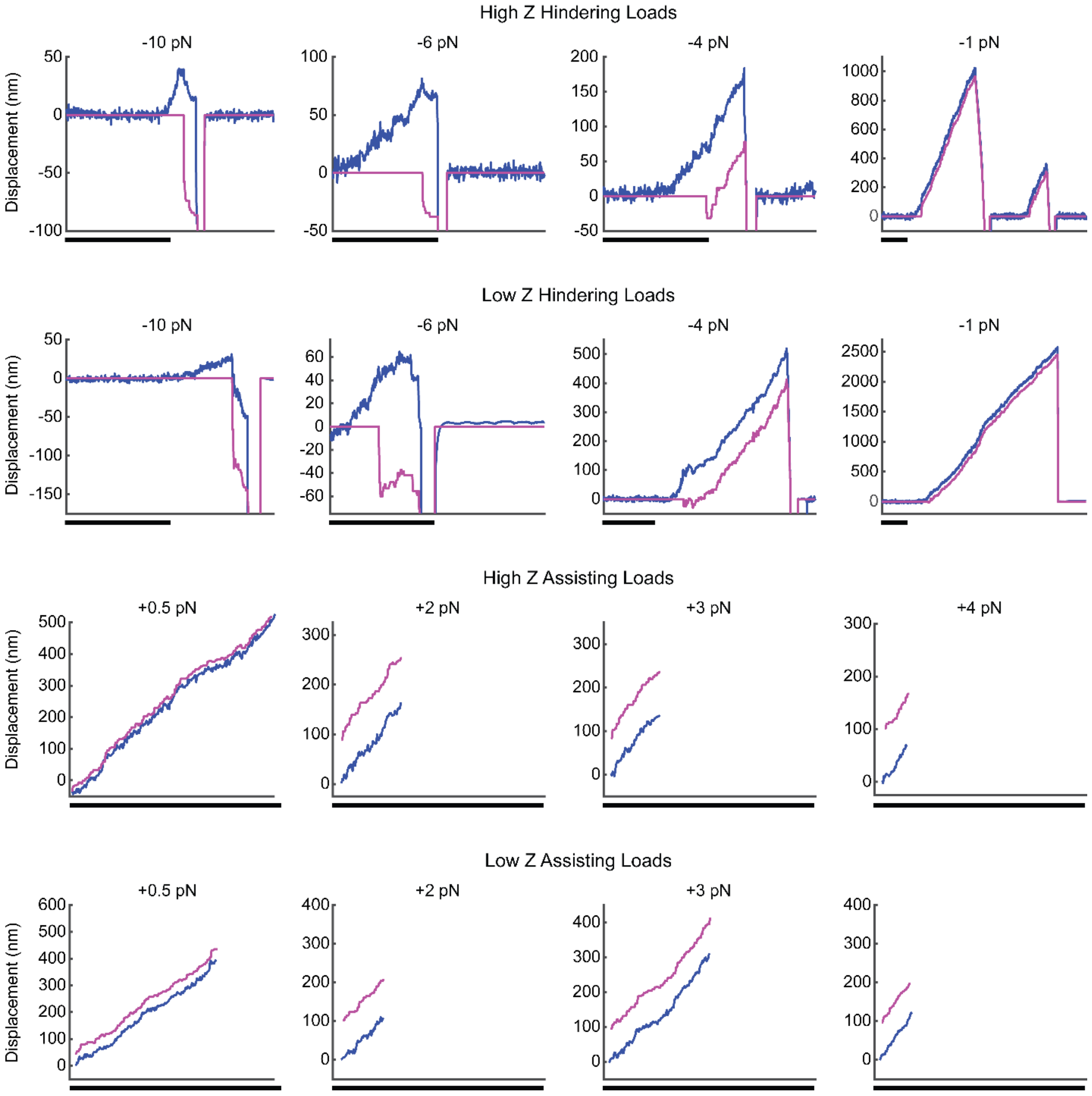

Figure 2_figure supplement 1. Example traces for force-feedback controlled trapping of kinesin with or without a DNA handle. Example traces for force feedback measurements under the high z-force and low z-force conditions in hindering and assisting directions. Scale bars are 1 s.

bioRxiv preprint doi: https://doi.org/10.1101/2025.08.27.672644; this version posted November 22, 2025. The copyright holder for this preprint (which was not certified by peer review) is the author/funder, who has granted bioRxiv a license to display the preprint in perpetuity. It is made available under aCC-BY 4.0 International license.

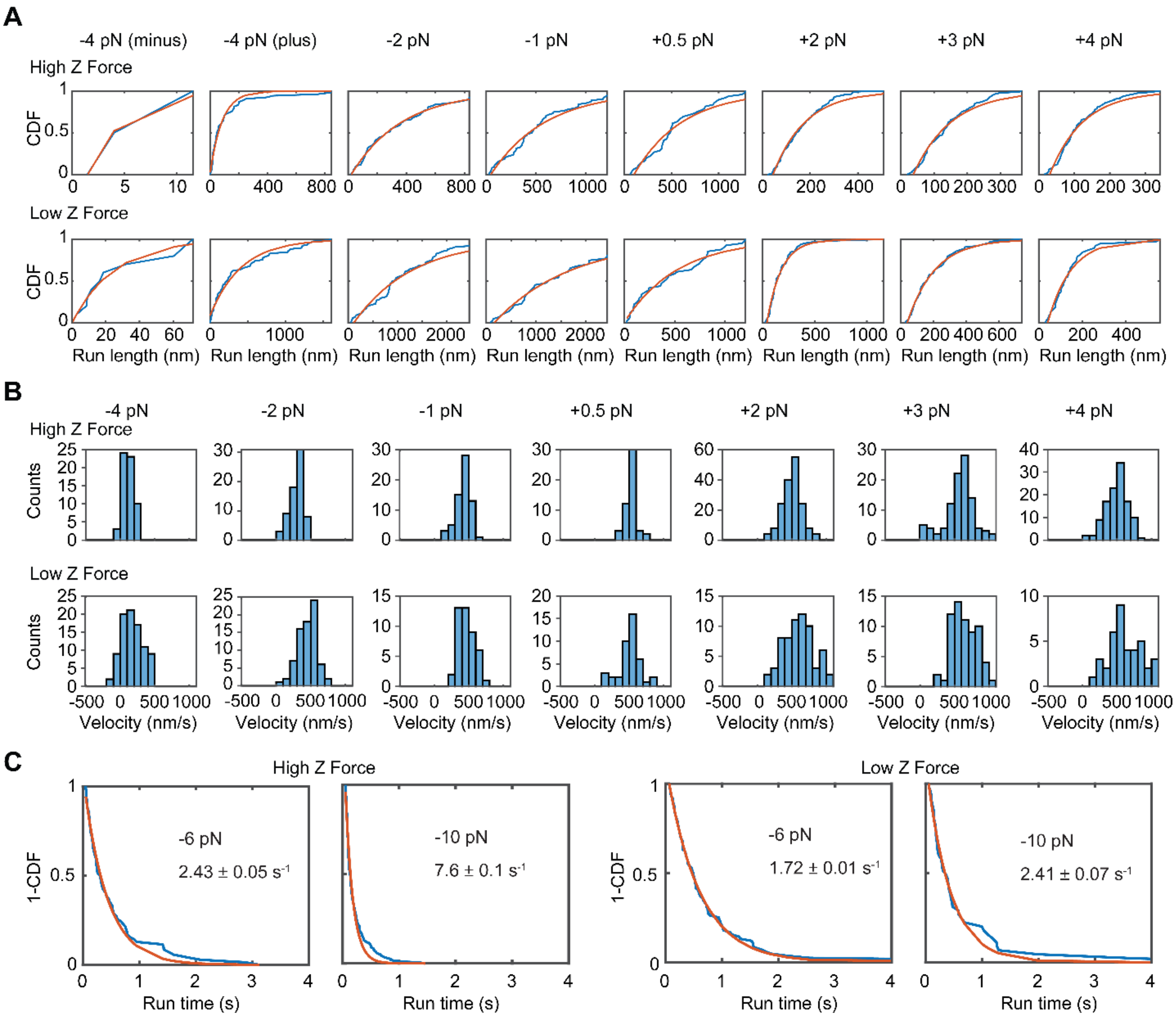

Figure 2_figure supplement 2. Raw histograms and fits to force-feedback controlled trapping of kinesin with or without a DNA handle. A) CDFs of run length at each condition (blue) and the corresponding fit (red). Under the 4 pN hindering condition, a small number of events demonstrated net negative run lengths. A weighted average was used to calculate the run length shown in Fig. 2. From left to right, N = 3, 55, 69, 65, 50, 167, 100, 115 on the top row, and 11, 78, 76, 44, 42, 65, 32, 39 in the bottom row. **B)** Histograms of velocities for each condition. From left to right, N = 60, 69, 65, 50, 167, 100, 115 in the top row and 89, 76, 44, 42, 65, 32, 39 in the bottom row. **C)** 1-CDFs of motor attachment time to the microtubule without net positive displacement under stall or superstall forces. Solid red curves represent a fit to a single exponential decay to calculate the detachment rate (From left to right, N = 167, 133, 73, 43).

bioRxiv preprint doi: https://doi.org/10.1101/2025.08.27.672644; this version posted November 22, 2025. The copyright holder for this preprint (which was not certified by peer review) is the author/funder, who has granted bioRxiv a license to display the preprint in perpetuity. It is made available under aCC-BY 4.0 International license.

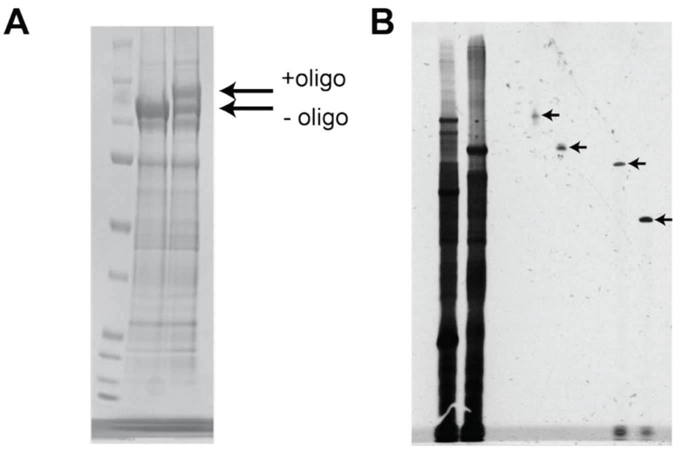

Figure 3_figure supplement 1. Purification and stall force of multi-motor chassis. A) SDS-PAGE denaturing gel to quantify oligo labeling of K560-GFP-SNAP. Left lane: molecular weight marker. Middle lane: K560-GFP-SNAP. Right Lane: K560-GFP-SNAP labeled with DNA oligo. Arrows show that the oligo-labeled motor is discernible from the unlabeled motor on the gel. **B)** Gel extraction of multi-motor chassis. The left two lanes show 3- and 2-motor chassis for high z-force measurements before gel extraction. The middle two lanes show 3- and 2-motor chassis for high z-force measurements after gel extraction. The right two lanes show 3- and 2-motor chassis for low z-force measurements after gel extraction.

bioRxiv preprint doi: https://doi.org/10.1101/2025.08.27.672644; this version posted November 22, 2025. The copyright holder for this preprint (which was not certified by peer review) is the author/funder, who has granted bioRxiv a license to display the preprint in perpetuity. It is made available under aCC-BY 4.0 International license.

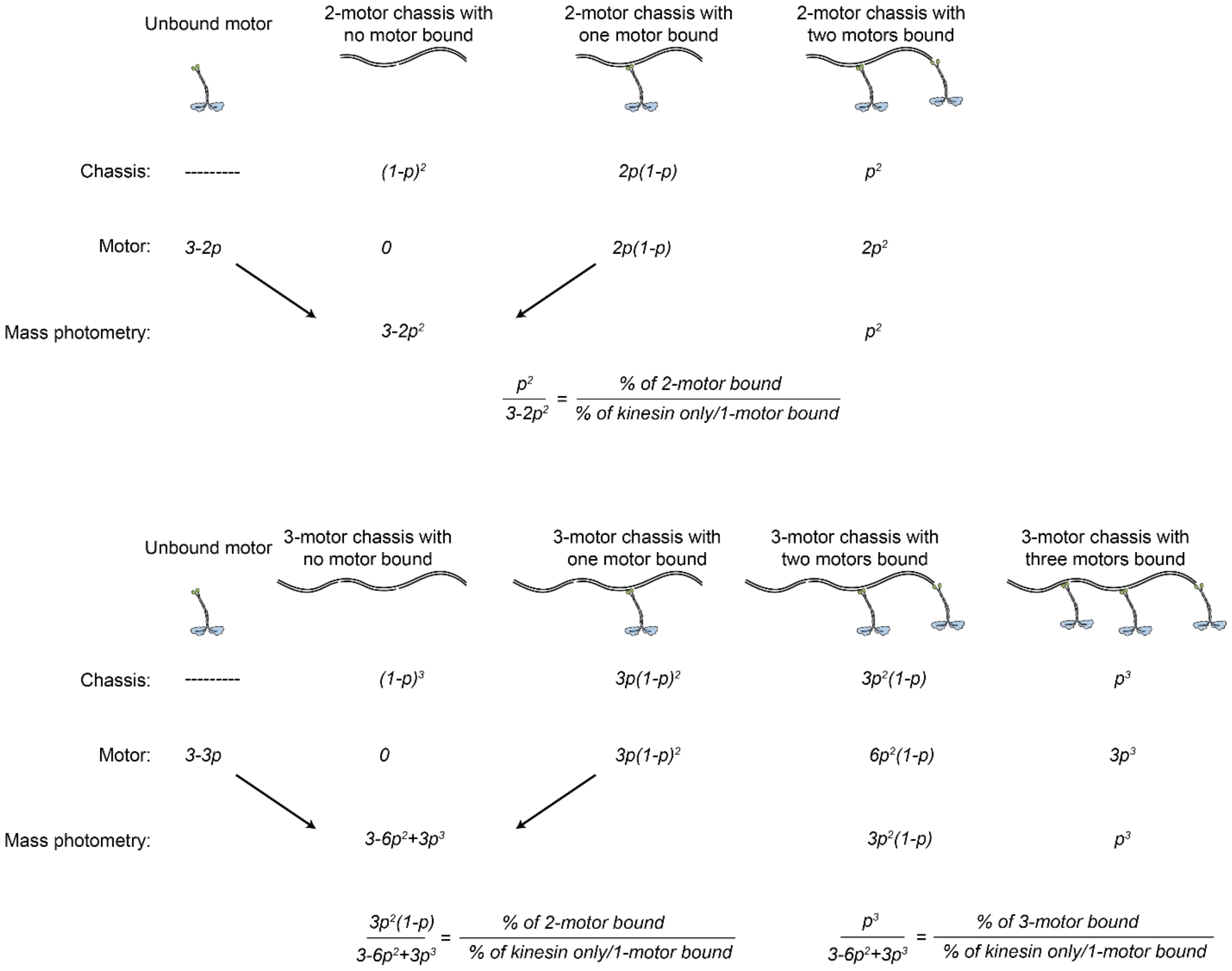

Figure 3_figure supplement 2. Model for estimating the percentage of DNA chassis bound to two- or three-motors in mass photometry. Unbound motor is 3-fold excess of the DNA chassis. p is the probability of binding of kinesin to the DNA chassis. The model assumes no cooperativity between the binding sites on the chassis. Motor bound to chassis was calculated from the probability of chassis multiplied by the number of motors bound to the chassis. Mass photometry cannot distinguish an unbound motor and chassis with one motor bound. p-values were calculated from the ratios of the percentages of distinct mass populations detected by mass photometry.

bioRxiv preprint doi: https://doi.org/10.1101/2025.08.27.672644; this version posted November 22, 2025. The copyright holder for this preprint (which was not certified by peer review) is the author/funder, who has granted bioRxiv a license to display the preprint in perpetuity. It is made available under aCC-BY 4.0 International license.

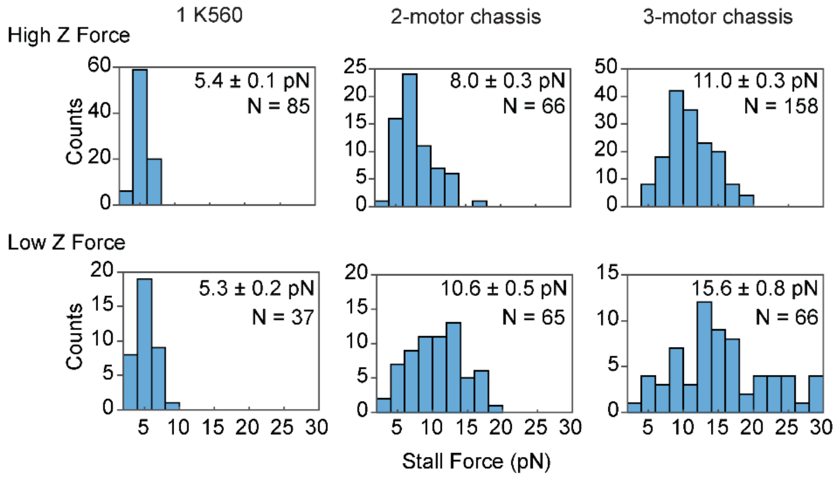

Figure 3_figure supplement 3. Stall force histograms (mean ± s.e.) of K560, and 2- or 3-motor chassis under high and low z-force conditions.

bioRxiv preprint doi: https://doi.org/10.1101/2025.08.27.672644; this version posted November 22, 2025. The copyright holder for this preprint (which was not certified by peer review) is the author/funder, who has granted bioRxiv a license to display the preprint in perpetuity. It is made available under aCC-BY 4.0 International license.

Materials and Methods

Construction of a Long DNA Handle

A PCR reaction was performed with 2 µl of 500 µg/mL lambda phage DNA (NEB), 15 µl each of Bio Forward and Kinesin Reverse primers (Table 1), 2.5 mL of KOD Hot Start Master Mix (NEB), and nuclease-free water up to 5 mL. This volume was subdivided into 96 tubes, and the PCR reaction was performed with annealing the primers at 57 ºC for 10 s and extension at 70 ºC for 85 s for 40 cycles. The resulting product was cleaned up using a PCR purification kit (Qiagen) and subsequently digested with BstXI for 2 h at 37 ºC followed by heat inactivation of the enzyme at 80 ºC for 20 min. The DNA handle was run on a 0.8% TAE agarose gel and the digested band was recovered via gel extraction. Oligos Kinesin 24B Bottom and 45B Top (Table 1) were annealed at 95 ºC for 2 min in annealing buffer (10 mM Tris, 50 mM NaCl, 5 mM EDTA, pH 8) and gradually cooled to room temperature over 1 h. The annealed oligos were then ligated onto the digested DNA handle by incubating with T4 polynucleotide kinase and T4 DNA ligase at ambient temperature overnight in the T4 DNA ligase reaction buffer. Excess oligo was removed with a PCR purification kit.

The presence of the single-stranded overhang was confirmed by hybridizing the handle with excess Cy3-labeled complementary oligomers (Table 1) and running it on an agarose gel. The gel was subsequently imaged in the Cy3 channel of a Typhoon FLA 9500 (Cytiva) to verify colocalization of the Cy3 oligo with the handle (Figure 1_figure supplement 1B).

Construction of the DNA Chassis

Four versions of the DNA chassis were used for multiple motor experiments. For the high z-force geometry, splint and backbone oligos were used as described previously (Elshenawy et al., 2019). The 2-motor chassis used the following oligos: M1-Tag-Bio, S1-Tag Splint, M1-S1, S1-2 Splint, M2-S1, whereas the 3-motor chassis used two additional oligos: S2-3 Splint and M3-S1 (Table 1). Briefly, 70 µl of biotinylated oligo was added to 50 µl backbone and 100 µl splint DNA oligos for the relevant constructs, annealed at 95 °C for 2 h, and gradually cooled to room temperature over 1 h. The resulting product was run on a 10% TBE gel (Cytiva). Bands containing the fully assembled chassis were extracted from the gel, flash frozen in liquid nitrogen, and crushed with a metal spatula. The crushed gel slices were then nutated in the crush and soak buffer (300 mM NaOAc, 1 mM EDTA, 0.1% SDS, pH 8) at room temperature for 48 h. After centrifugation at 5,000 g for 2 min, the supernatant was collected and passed through a DNA spin column (EconoSpin) to remove remaining polyacrylamide. The DNA was further purified using ethanol precipitation and resuspended in 10 mM Tris, pH 8. The concentration was determined from the absorbance at 260 nm using a Nanodrop 1000 (ThermoFisher). For reduced z-force experiments, long ultramer oligos were ordered from IDT (Table 1) and diluted in 10 mM Tris pH 8 for a final concentration of 100 µM.
