# Supplementary material for: Enhanced Processivity and Collective Force Production of Kinesins at Low Radial Forces
Source: bioRxiv. 2025 Aug 31:2025.08.27.672644. Preprint. [Version 1] doi: 10.1101/2025.08.27.672644 (PMC12407981; doi:10.1101/2025.08.27.672644)
Supplement: Supplementary file 1 [file NIHPP2025.08.27.672644v1-supplement-1.pdf]

bioRxiv preprint doi: https://doi.org/10.1101/2025.08.27.672644; this version posted August 31, 2025. The copyright holder for this preprint (which was not certified by peer review) is the author/funder, who has granted bioRxiv a license to display the preprint in perpetuity. It is made available under aCC-BY 4.0 International license.

Supplementary Figures

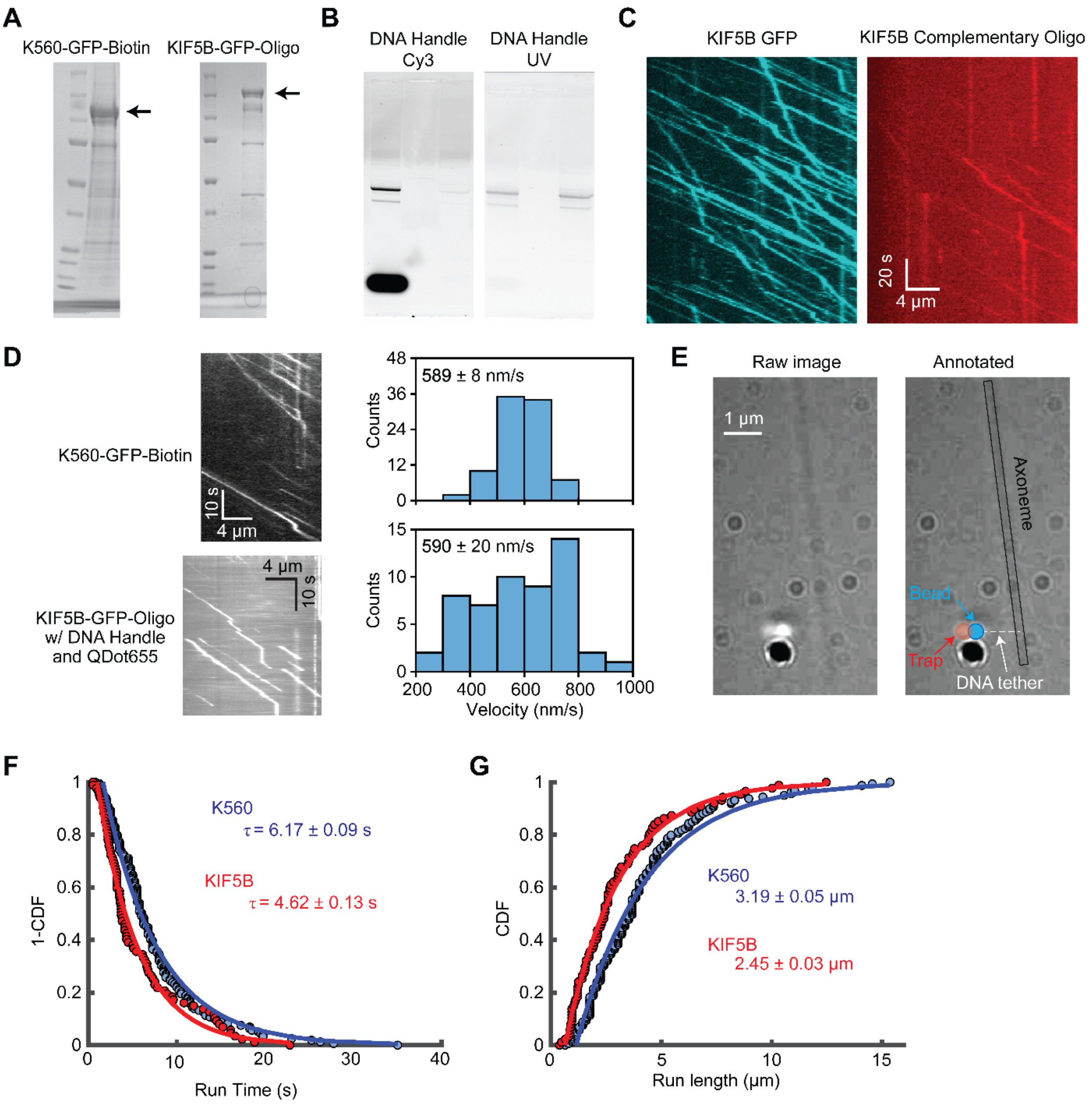

Figure 1_figure supplement 1. Purification of kinesin and DNA handle with experimental controls. A) SDS-Page denaturing gel of constructs used. K560-GFP-SNAP was labeled with biotin, and KIF5B-GFP-SNAP was labeled with a BG-functionalized DNA oligo before eluting the motor from the IgG beads during purification. **B)** The long DNA handle was run on a 0.8% TAE agarose gel. Left: Handle incubated with a 20-fold excess of complementary Cy3 oligo and imaged in the Cy3 channel of a Typhoon FLA 9500 fluorescence imager. Right: The same gel imaged under UV after staining in 2x GelRed for 40 minutes. **C)** Full-length KIF5B-GFP-SNAP was incubated with a 10-fold excess of complementary Cy5-labeled oligo and run in a motility assay. The assay was performed in the presence of 10 nM MAP7. **D)** Kymographs of K560-GFP-

bioRxiv preprint doi: https://doi.org/10.1101/2025.08.27.672644; this version posted August 31, 2025. The copyright holder for this preprint (which was not certified by peer review) is the author/funder, who has granted bioRxiv a license to display the preprint in perpetuity. It is made available under aCC-BY 4.0 International license.

biotin (top) and Qdot 655 streptavidin quantum dots being transported by oligo-labeled KIF5B attached to the long DNA handle (bottom). Histograms of the respective velocities are shown to the right. **E)** Mechanical demonstration of a bead tethered to an axoneme by the long DNA handle. After tethering to an axoneme, the bead is pulled away from the axoneme by an optical trap. The bead moved freely for ~1 μm and stayed in that position, demonstrating the formation of a long tether between the bead and the axoneme through the DNA handle. **F)** 1-CDF kinesin run time under unloaded conditions in single-molecule fluorescence imaging assays. Solid curves represent a fit to a single exponential decay function to calculate the lifetime of processive runs (τ, ±S.E.). **G)** CDF of kinesin run length under unloaded conditions in single-molecule fluorescence imaging assays. Solid curves represent a fit to a single exponential decay function to calculate the mean run length (±S.E.). In D, F, and G, KIF5B assays were performed in the presence of 50 nM MAP7.

bioRxiv preprint doi: https://doi.org/10.1101/2025.08.27.672644; this version posted August 31, 2025. The copyright holder for this preprint (which was not certified by peer review) is the author/funder, who has granted bioRxiv a license to display the preprint in perpetuity. It is made available under aCC-BY 4.0 International license.

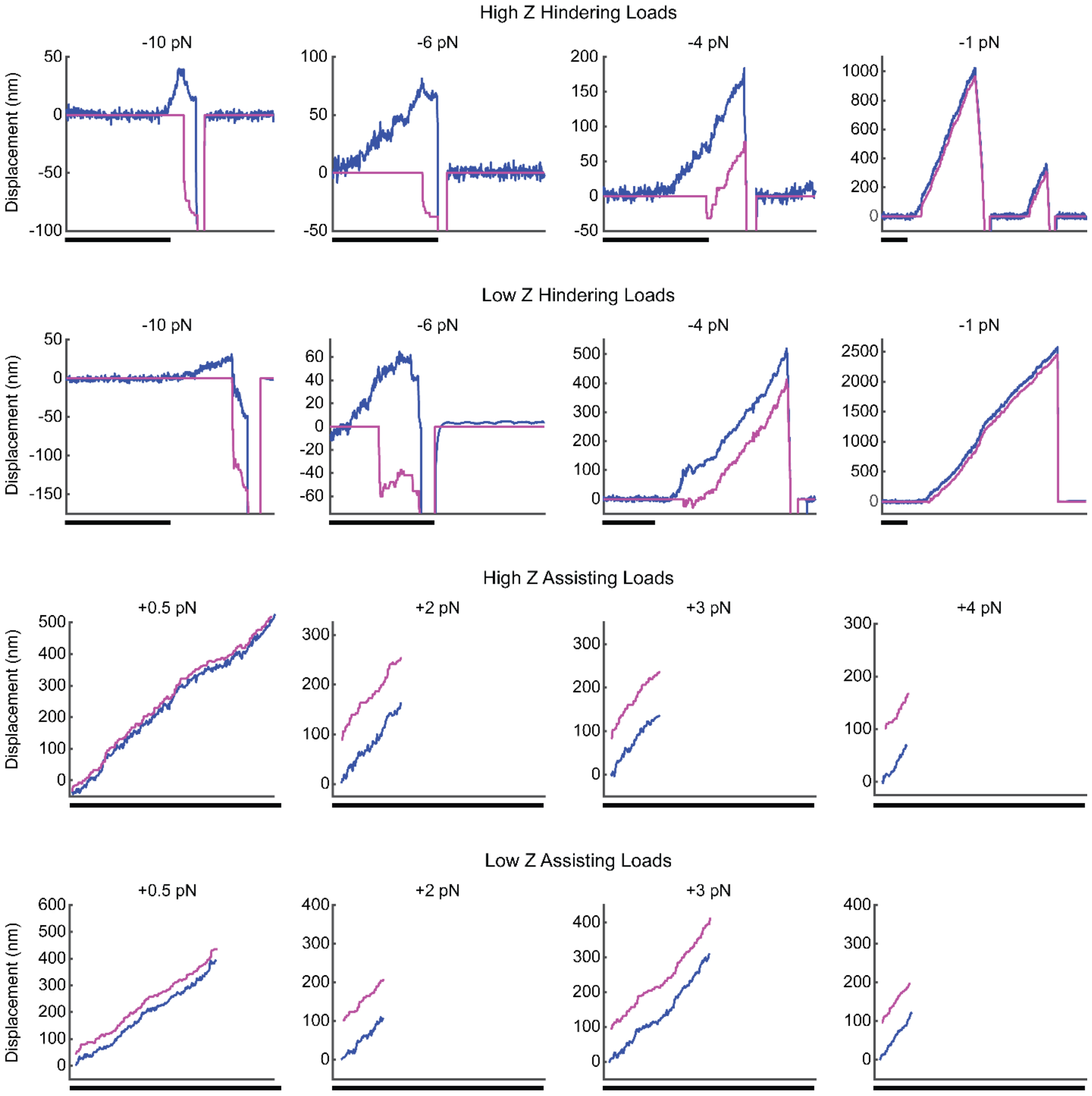

Figure 2_figure supplement 1. Example traces for force-feedback controlled trapping of kinesin with or without a DNA handle. Example traces for force feedback measurements under the high z-force and low z-force conditions in hindering and assisting directions. Scale bars are 1 s.

bioRxiv preprint doi: https://doi.org/10.1101/2025.08.27.672644; this version posted August 31, 2025. The copyright holder for this preprint (which was not certified by peer review) is the author/funder, who has granted bioRxiv a license to display the preprint in perpetuity. It is made available under aCC-BY 4.0 International license.

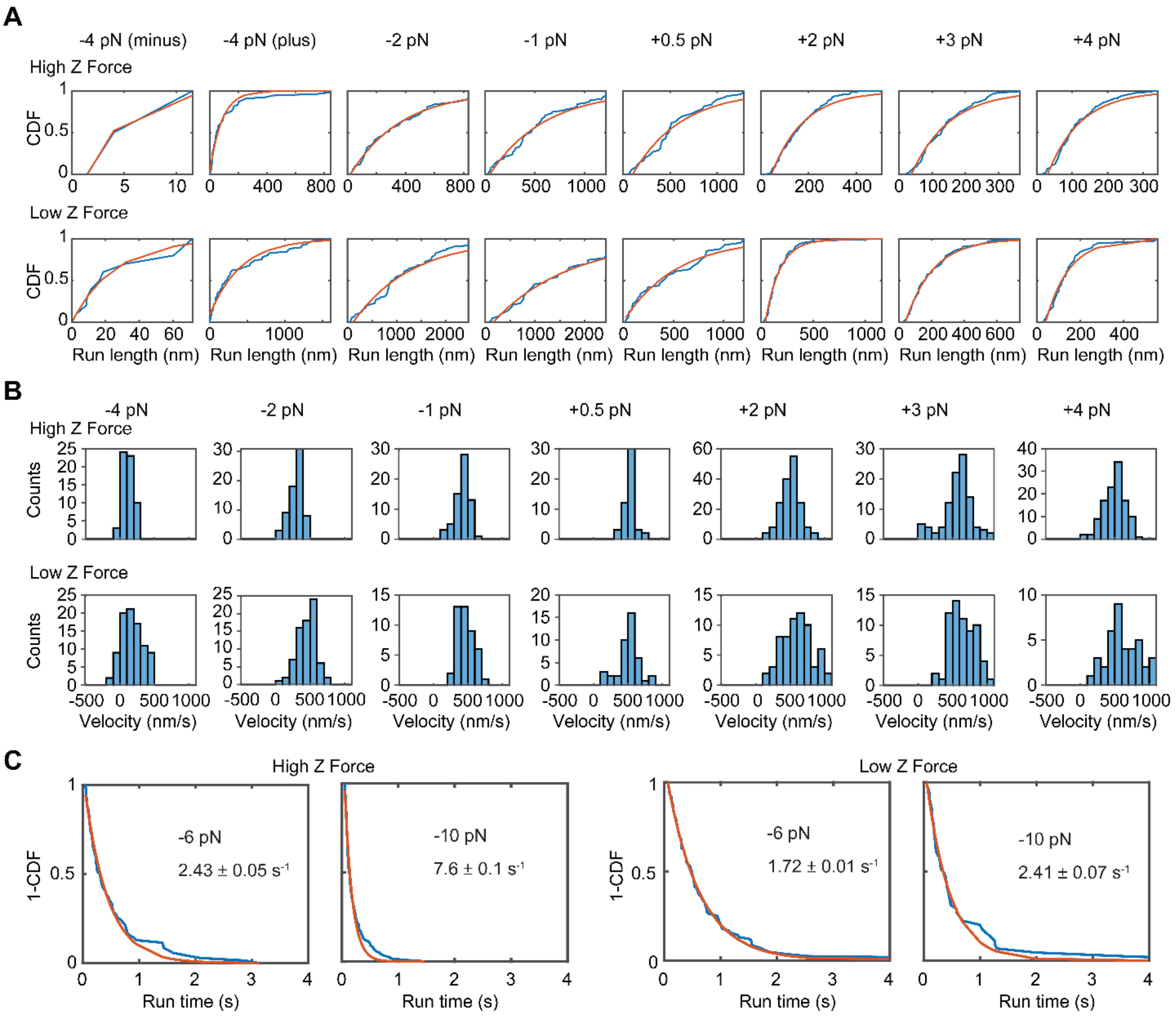

Figure 2_figure supplement 2. Raw histograms and fits to force-feedback controlled trapping of kinesin with or without a DNA handle. A) CDFs of run length at each condition (blue) and the corresponding fit (red). Under the 4 pN hindering condition, a small number of events demonstrated net negative run lengths. A weighted average was used to calculate the run length shown in Fig. 2. **B)** Histograms of velocities for each condition. **C)** 1-CDFs of motor attachment time to the microtubule without net positive displacement under stall or superstall forces. Solid red curves represent a fit to a single exponential decay to calculate the detachment rate.

bioRxiv preprint doi: https://doi.org/10.1101/2025.08.27.672644; this version posted August 31, 2025. The copyright holder for this preprint (which was not certified by peer review) is the author/funder, who has granted bioRxiv a license to display the preprint in perpetuity. It is made available under aCC-BY 4.0 International license.

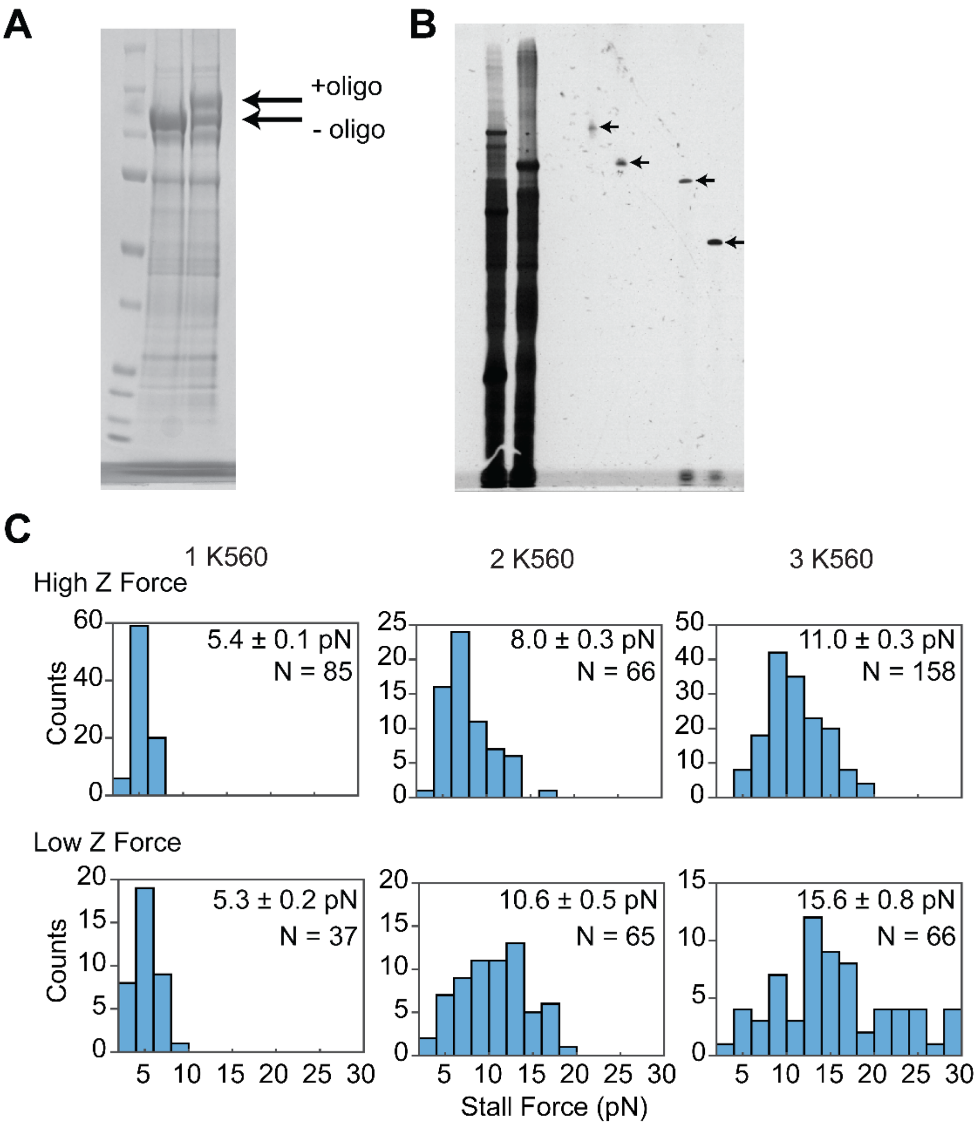

Figure 3_figure supplement 1. Purification and stall force of multi-motor chassis. A) SDS-PAGE denaturing gel to quantify oligo labeling of K560-GFP-SNAP. Left lane: molecular weight marker. Middle lane: K560-GFP-SNAP. Right Lane: K560-GFP-SNAP labeled with DNA oligo. Arrows show that the oligo-labeled motor is discernible from the unlabeled motor on the gel. **B)** Gel extraction of multi-motor chassis. The left two lanes show 3- and 2-motor chassis for high z-force measurements before gel extraction. The middle two lanes show 3- and 2-motor chassis for high z-force measurements after gel extraction. The right two lanes show 3- and 2-motor chassis for low z-force measurements after gel extraction. **C)** Stall force histograms (mean ± s.e.) of one, two, or three motor chassis under high and low z-force conditions.
